# Myoferlin, a Membrane Protein with Emerging Oncogenic Roles

**DOI:** 10.1155/2019/7365913

**Published:** 2019-11-19

**Authors:** Yimin Dong, Honglei Kang, Huiyong Liu, Jia Wang, Qian Guo, Chao Song, Yunlong Sun, Ya Zhang, Honghua Zhang, Zheng Zhang, Hanfeng Guan, Zhong Fang, Feng Li

**Affiliations:** ^1^Department of Orthopedic Surgery, Tongji Hospital, Tongji Medical College, Huazhong University of Science and Technology, Wuhan, China; ^2^Biological Engineering and Regenerative Medicine Center, Tongji Hospital, Tongji Medical College, Huazhong University of Science and Technology, Wuhan, China

## Abstract

Myoferlin (MYOF), initially identified in muscle cells, is a member of the Ferlin family involved in membrane fusion, membrane repair, and membrane trafficking. Dysfunction of this protein is associated with muscular dysfunction. Recently, a growing body of studies have identified MYOF as an oncogenic protein. It is overexpressed in a variety of human cancers and promotes tumorigenesis, tumor cell motility, proliferation, migration, epithelial to mesenchymal transition, angiogenesis as well as metastasis. Clinically, MYOF overexpression is associated with poor outcome in various cancers. It can serve as a prognostic marker of human malignant disease. MYOF drives the progression of cancer in various processes, including surface receptor transportation, endocytosis, exocytosis, intercellular communication, fit mitochondrial structure maintenance and cell metabolism. Depletion of MYOF demonstrates significant antitumor effects both in vitro and in vivo, suggesting that targeting MYOF may produce promising clinical benefits in the treatment of malignant disease. In the present article, we reviewed the physiological function of MYOF as well as its role in cancer, thus providing a general understanding for further exploration of this protein.

## 1. Introduction

MYOF is a member of the Ferlin family that has six Ferlin proteins, Fer-1, Sea urchin Ferlin, Misfire, Otoferlin, MYOF, and dysferlin [1]. Fer-1, Sea urchin Ferlin and Misfire are reported in C. elegans, Sea urchin, Drosophila, respectively. Dysfunction of these proteins is associated with infertility or defective exocytosis [2–4]. Otoferlin, MYOF, and dysferlin are mainly studied in human tissues. Otoferlin exists in the hair cells of the inner ear. Mutation of its gene causes a nonsyndromic prelingual deafness [5]. Dysferlin is highly expressed in human muscular tissues and mediates membrane repair in a Ca^2+^-dependent manner [6, 7]. Loss of dysferlin is associated with two types of human muscular dystrophy, Miyoshi myopathy and limb girdle muscular dystrophy [8, 9]. Interestingly, MYOF also abounds in human muscular tissue. It is important for membrane repair and myoblast fusion. MYOF-null myoblasts can enter into the initial stage of fusion events, but they lose the ability to form large maturate myofibers and to regenerate [10].

MYOF and dysferlin seem to have overlapping roles in skeletal muscles and they may interact with each other [11]. Compared to dysferlin-null mice, mice lacking both myoferlin and dysferlin develop severer muscular damage [12], and transgenic overexpression of MYOF attenuates membrane fusion defects in muscle cells of dysferlin-deficient mice [13]. However, MYOF is mainly located in immature “prefusion” myoblasts, while dysferlin exhibits abundance in mature myotubes [14]. In limb-girdle muscular dystrophy type 2B caused by dysferlin mutation, no difference in muscular MYOF protein level has been observed between mildly and severely affected patients [15]. In dysferlinopathy, compensatory overexpression of myoferlin is absent in affected muscles [16]. These findings indicate that MYOF can partially compensate for the function of dysferlin, but this compensation does not occur naturally as a rescue mechanism in the absence of dysferlin.

While dysferlin is mainly found in muscle tissue, MYOF has been identified in many other tissues and cells, including human airway epithelia [17], human placenta [18, 19], vascular tissues and endothelial cells (ECs) [20]. Recently, increasing studies have revealed that MYOF is overexpressed in many kinds of human cancers, including pancreas cancer [21, 22], ovarian carcinoma [23], prostate cancer [24], breast cancer and renal cell cancer [25, 26], indicating that this protein may have important roles in tumorigenesis and malignant progression. However, the exact role of MYOF in tumorigenesis has not been fully elucidated yet. In the present article, we reviewed the structure and physiological function of MYOF, and summarized the role of MYOF in malignant disease, thus providing a general understanding of this protein.

## 2. The Structure of MYOF

**Figure 1:**
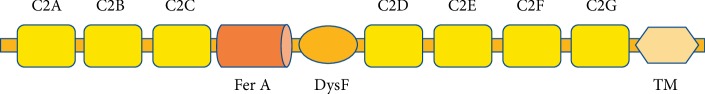
Schematic structure of MYOF. MYOF has seven C2 domains, a Ferlin A domain, a DysF domain as well as a single C-terminal transmembrane helix. C2-domains are responsible for Ca^2+^-dependent lipid binding and interacting with targeting proteins. FerA binds to phospholipids in a Ca^2+^ independent manner. DysF is possibly responsible for receptor-mediated endocytosis.

All six ferlins consist of multiple C2-domains, a centrally-positioned FerA domain and a single C-terminal transmembrane helix. Like other Ferlins, typical structure of MYOF is multiple C2-domains that regulate the protein's function by forming Ca^2+^-dependent phospholipid complex [27]. MYOF has seven C2-domains (Figure 1). These C2-domains can bind to lipid layers and change the structure of lipid packing, forming regional distorted membrane structure responsible for fusion and fission events [28]. C2-domains are surrounded by a ring of positive charges. Ca^2+^-binding to C2-domains causes a major change in the electrostatic potential and mediates binding with target molecules [29]. C2-domains of MYOF have equivalent Ca^2+^-binding affinity, which is weaker than that of Dysferlin, however [30]. FerA domain is a four-Helix bundle domain associated with the membrane. MYOF FerA domain preferably binds to negatively-charged phospholipids. This domain has no Ca^2+^-binding site, but its binding activity can be enhanced by Ca^2+^ [31]. MYOF and dysferlin have additional DysF domain than other Ferlin proteins (Figure 1). DysF domain of MYOF can bind to caveolin-3, a protein that functions as a component of the caveolae plasma membrane found in most cell types [32].

## 3. MYOF Is Involved in a Wide Range of Membrane Trafficking Events

**Figure 2:**
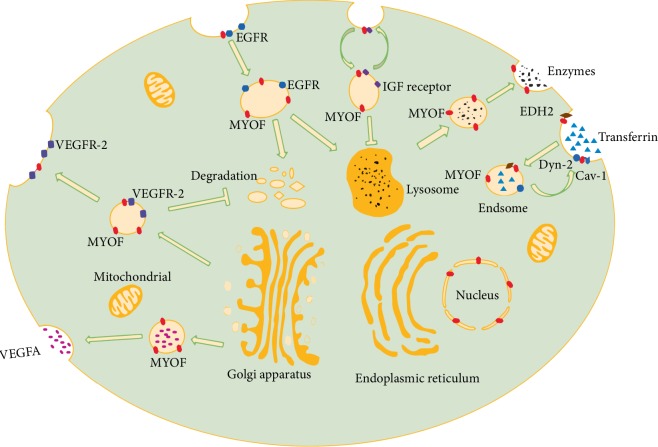
The role of MYOF in membrane trafficking. These trafficking processes have been identified in muscle fibers and other cells. They are also present in cancer cells. MYOF is present at the plasma and nucleus membrane as well as intracellular vesicle. It is involved in exocytosis, endocytosis, receptor internalization and recycling. For example, MYOF mediates endocytosis of transferrin by interacting with caveolin-1 (Cav-1) and dynamin-2 (Dyn-2). MYOF also directs vesicles containing VEGFR-2 to the plasma membrane to promote its surface expression and prevents it from proteasomal degradation. In addition, MYOF is responsible for internalization and recycling of receptors like IGF receptor. MYOF depletion redirects IGF receptor from recycling to a degradation pathway, which leads to mistrafficking of such receptor and disrupts IGF signaling. The secretion of lysosomal enzymes also requires MYOF, suggesting that this protein is widely involved in membrane trafficking.

MYOF is expressed in various membrane structures, including the plasma membrane, perinuclear vesicular puncta, Rab7-positive endosomes as well as cell periphery [33], indicating that it participates in a wide range of membrane trafficking processes (Figure 2). Doherty et al. have reported that MYOF participates in endocytic trafficking by interacting with EH domain containing protein 2 (EHD2) [34]. EHD proteins, including EHD1-4, are associated with endocytic transport such as cell surface receptor internalization and recycling [35, 36]. MYOF depletion decreases EHD2 level and delays recycling of foreign protein such as transferrin, resulting in delayed recycling and intracellular accumulation of transferrin after its internalization [34]. In myoblasts, EHD1 is co-localized with MYOF, forming a prefusion complex that is directed to surface membrane by the Rho-GAP, GRAF1 (GTPase regulator associated with focal adhesion kinase-1). Knockdown of any of these three proteins can impair myoblast fusion, suggesting a multiple, not single protein involvement in MYOF-mediated membrane fusion events [37, 38].

MYOF also participates in recycling of insulin-like growth factor (IGF) receptor (Figure 2), which initiates an important signaling for normal myogenesis. MYOF depletion stalls IGF receptor recycling to the plasma membrane and redirects it toward lysosomal degradation, resulting in hampered IGF receptor related pathway as well as unresponsiveness to IGF1 stimulation both in vitro and in vivo [39]. In endothelial cells (ECs), MYOF forms a complex with vascular endothelial growth factor receptor 2 (VEGFR-2) and mediates its membrane-oriented expression [20]. MYOF prevents VEGFR-2 from polyubiquitination as well as proteasomal degradation (Figure 2). Loss of MYOF reduces VEGF-mediated activation of key intracellular signaling cascades, such as ERK-1/2, JNK, and PLC*γ*. Another EC-specific angiogenic receptor of angiopoietin-1, Tie-2, also necessities MYOF for normal membrane localization [40]. MYOF silencing exhibits significant antiangiogenetic effects, indicating that it is a potential antiangiogenic target and targeting this protein may yield potential therapeutic benefits in angiogenesis-related disease.

Another study has revealed that MYOF participates in caveolae/lipid raft and clathrin-mediated endocytosis by forming a complex with dynamin-2 (Dyn-2) and caveolin-1 (Cav-1) [41]. Dyn-2 is essential for the fission process of endocytic vesicle, while Cav-1 is a structural component of caveolae. The MYOF/Dyn-2/Cav-1 complex is required for endocytosis and membrane repair. Respective loss of the three proteins impairs endocytosis of transferrin and leads to equal loss of membrane resealing following membrane injury [41, 42]. In addition to endocytosis, a recently published study has uncovered the role of MYOF in lysosomal exocytosis of phagocytes [43]. MYOF mediates fusion of exocytotic lysosome to the membrane and helps to release lysosome contents, including hydrolytic enzymes, which increase cytotoxicity (Figure 2). It is possible that MYOF is involved in phagocyte-mediated inflammation and antitumor effects by enhancing its cytotoxicity effects.

Schiller et al. have reported that MYOF is also present at extracellular membrane structures [44] and interacts with leukocyte specific transcript 1 (LST1), a transmembrane MHC class III protein that can induce the formation of tunneling nanotubes (TNT) essential for cell-to-cell communication [45]. MYOF is speculated to drive LST1-induced TNT formation by locally deforming the plasma membrane and mediating membrane fusion [44]. Blomme et al. have identified the expression of MYOF in tumor cell derived exosomes. MYOF depletion decreases the ability of such exosomes to induce migration and proliferation of human endothelial cells [46]. However, further studies are required to elucidate the exact role of MYOF in extracellular structures in different cells and tissues and to explore its association with human disease.

## 4. Role of MYOF in Cancer

As reviewed above, MYOF is implicated in a wide range of membrane-associated events. It is likely that cancer cells also replicate the role of MYOF to facilitate their progression because they are highly active in membraneassociated events to maintain a malignant phenotype. Recently, emerging studies have revealed that MYOF is overexpressed in many cancers. Overexpression of MYOF is associated with poor prognosis in patients with breast cancer, lung cancer, and pancreas cancer [47, 48]. In pancreas adenocarcinoma (PAC), MYOF expression is negatively correlated with the degree of histological differentiation and patients with MYOF-overexpressing MYOF have poor total survival rate after surgical treatment. In addition, MYOF has been found to have recurrent mutations which are associated with the clinical outcome of patients with non-small cell lung cancer [49]. In oropharyngeal squamous cell carcinoma (OPSCC), nuclear location of MYOF is significantly related to poor prognosis, as patients with nuclear MYOF+/IL-6+OPSCC have the worst, whereas nuclear MYOF−/IL-6−OPSCC have the best outcome [50]. However, this is not the same case in all cancers. In endometrioid carcinoma, moderate to strong expression of MYOF is observed in normal epithelial tissue and low-grade carcinoma tissue, whereas weak to negative expression is found in high-grade carcinoma tissue [51]. MYOF contributes to tumorigenesis in various processes, and the mechanisms are discussed as follows based on available data.

### 4.1. MYOF in Breast Cancer

MYOF has been intensively studied in breast cancer, which is one of the most common cancers worldwide and represents the leading cause of cancer related female death [52]. In human breast cancer cells, MYOF regulates cell-matrix adhesion by affecting the strength of focal adhesion, structure that plays a crucial role in cell migration [53] and matrix degradation [54]. MYOF depletion increases focal adhesion maturity and cell-substrate adhesion, which renders tumor cells a more epithelial-like morphology [55]. Another study has reported that MYOF increases cell migration by functioning in the postligand pathway of epidermal growth factor receptor (EGFR) [56]. MYOF-silencing impedes the proteasomal and lysosomal degradation of cargo containing EGFR (Figure 2), leading to intracellular accumulation of EGFR and continuous activation of phosphorylated EGFR (p-EGFR) [56]. Increased intracellular EGFR has detrimental effects on cells [57], which causes unresponsiveness of tumor cells to EGF stimulation and induces apoptosis.

Other studies have revealed that MYOF depletion can stall invasion and reverse epithelial to mesenchymal transition (EMT) [58, 59]. Decreased cell invasion by MYOF silencing may partially result from down-regulation of several matrix metalloproteinases (MMPs), especially of MMP1, which plays a pivotal role in degrading ECM components to facilitate cancer invasion [59, 60]. EMT is characterized by downregulation of E-cadherin and upregulation of vimentin [61]. MYOF can reduce E-cadherin by enhancing the enzymatic activity of a disintegrin and metalloproteinase 12 (ADAM12), a metalloproteinase that is highly upregulated in multiple tumors [62]. MYOF depletion results in high levels of E-cadherin and low levels of fibronectin and vimentin, indicating that MYOF precipitates EMT to promote tumor invasion. Recently, a small molecule, WJ460, has been identified to target the C2-domain of MYOF. This molecule can reverse EMT of breast cancer and decrease pulmonary metastasis [63]. Another diaryl-1,2,4-triazole derived compound targeting MYOF also demonstrates potent antitumor effects similar to that of MYOF depletion [64]. Such MYOF-targeting compounds are expected to yield promising therapeutic benefits in the treatment of cancer.

Blomme et al. have described that MYOF is involved in lipid trafficking and mitochondrial function [65]. Intracellular transportation of lipids is blocked in cells deficient in MYOF, leading to increased accumulation of lipids and fatty acids, which impairs mitochondrial function and results in marked alteration of cell metabolism [65]. It is widely acknowledged that tumor cells tend to favor metabolism via glycolysis rather than oxidative phosphorylation which is termed as the Warburg effect [66]. However, accumulating evidence has revealed that they also tend to produce energy through oxidative phosphorylation to sustain survival and metastasis [67]. MYOF depletion decreases the oxidative phosphorylation (OXPHOS) as well as ATP production and redirects tumor cells toward glycolysis. However, this metabolic change renders tumor cells more susceptible to metabolism-targeting drugs, which might be a potential combined therapy for the prevention of tumor invasion [65].

### 4.2. MYOF in Digestive Organ Cancers

MYOF has also been explored in cancers of digestive organs, including pancreas, liver and colon. High expression of MYOF is associated with high density of blood vessels in pancreas cancer [68]. MYOF can facilitate secretion of vascular endothelial growth factor A (VEGFA) (Figure 2), a key factor for angiogenesis, while MYOF silencing impairs exocytosis of VEGFA by blocking the fusion process of VEGFA-containing vesicles with plasma membrane, which reduces VEGFA secretion from tumor cells and in return attenuates tumor-related angiogenesis [68]. MYOF also abounds in lipogenic pancreatic cancer cell lines and is important for mitochondrial fitness as well as energy production through OXPHOS. Contribution of MYOF to cancer cell migration is also OXPHOS-dependent in Pancreatic ductal adenocarcinoma [69]. MYOF depletion activates autophagy as a rescue mechanism in response to decreased energy production, but this rescue mechanism fails to restore proliferation to a controlled level [70]. It is possible that blocking autophagy may enhance the antitumor effects of MYOF depletion.

In colon cancer, MYOF silencing leads to accumulation of reactive oxygen species (ROS) and DNA damage. Increased ROS triggers p53 activation [71], which then provokes cell cycle arrest and results in p53-dependent reduction of cell growth. Reactivation of p53 can enhance the inhibition effects of MYOF-silencing on tumor cell growth, indicating that MYOF drives oncogenesis in colon cancer possibly by inhibiting the function of p53 [72]. It is anticipated that combination of MYOF-targeting and p53 modulation might yield new perspectives in the treatment of colon cancer.

**Figure 3:**
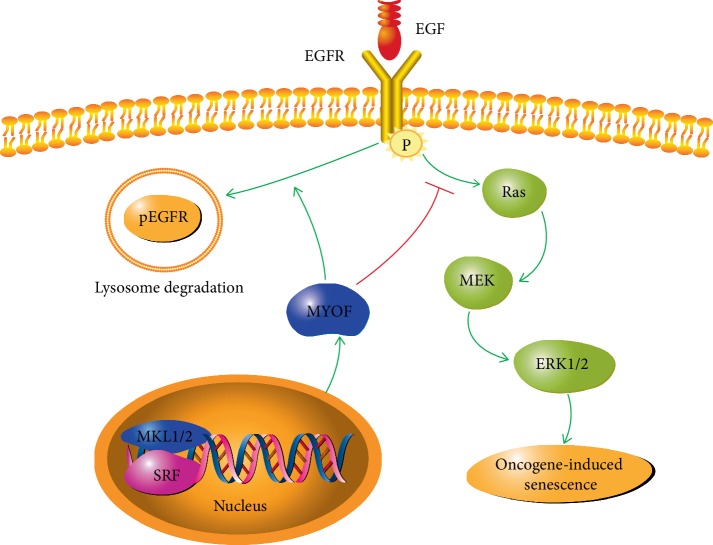
Role of MYOF in EGFR-related pathway. *MYOF* gene is induced by MKL1/2 and SRF. MYOF directs p-EGFR to lysosome degradation and shuts off EGFR related signaling. Inhibition of MYOF leads to continuous phosphorylated EGFR, which further activates Ras/MEK/ERK and results in Oncogene-induced senescence.

Most studies on MYOF in human cancer are mainly centred on its oncogenic functions, but little has been known about how MYOF gene expression is regulated. Previous study has shown that nuclear factor of activated T cells (NFAT) can bind to the promoter of MYOF gene and upregulate MYOF expression in fusing myoblasts and damaged MYOF fibers [73], but little is known about how MYOF gene expression is regulated in cancer cells. Hermann et al. have identified MYOF as a target gene downstream of the MLK1/2-SRF signaling axis in human and marine hepatocellular cancer cells (HCCs) [74]. Both MLK 1/2 and SRF are oncogenic drivers for hepatocellular cancer [75, 76]. MLK1/2 and SRF can bind to the promoter of MYOF gene and activate its expression. Either MKL1/2 or SRF depletion decreases MYOF level and triggers EGFR phosphorylation similar to that in breast cancer cells [56]. In HCCs, phosphorylated EGFR activates its downstream Ras/MEK/ERK cascade and induced oncogene-induced senescence (OIS) (Figure 3). As senescence induction is an emerging strategy for the treatment of HCC, it is possible that intervening in the MKL1/2—SRF-MYOF signaling pathway may contribute to the treatment of hepatocellular cancer [74].

### 4.3. Role of MYOF in Other Cancers

**Figure 4:**
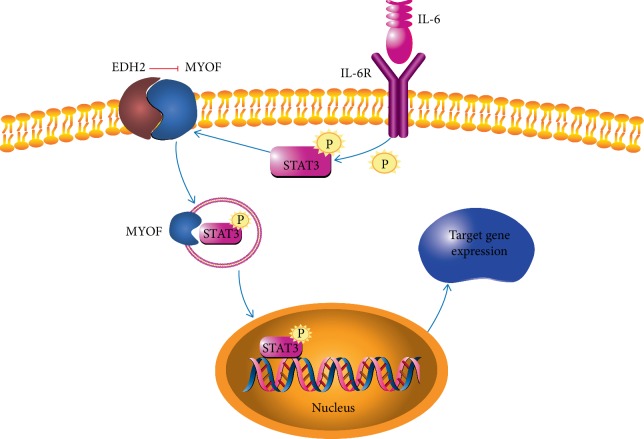
The role of MYOF in IL-6R related pathway. Activity of MYOF is inhibited by EDH2 on cell membrane. Upon stimulation by IL-6, IL-6R phosphorylates STAT3, which interacts with MYOF and translocates to the nucleus. Phosphorylated STAT3 activates its target gene expression and promotes tumor progression.

As an oncogenic protein, MYOF is ubiquitously expressed in human epithelial cancers. Study by Leung et al. has revealed that MYOF is overexpressed in Mouse Lewis lung carcinoma (LLC) cell. It is essential for plasma membrane integrity and LCC tumor cell proliferation [77]. In vivo, mice bearing LCC solid tumor exhibit significant reduction in tumor body in the absence of MYOF. In melanoma, MYOF contributes to the formation of Vasculogenic mimicry (VM), an important mechanism to facilitate tumor metastasis. MYOF depletion decreases exocytosis of MMP2 and VM formation, which further attenuates migration and invasion [78]. In the molecule level, MYOF serves as a chaperone protein in IL6/STAT3 signaling cascade and its activity is naturally suppressed by EHD2 in resting cells [79]. Upon the stimulation of IL-6, IL-6R triggers STAT3 phosphorylation and MYOF dissociation from EHD2. Then, phosphorylated STAT3 translocates to nucleus chaperoned by MYOF and activates downstream genes of the IL6/STAT3 pathway, thus potentiating tumor cell motility and cancer stem cell phenotype (Figure 4). In follicular lymphoma, MYOF has been identified as a self-antigen that can be recognized by surface BCR of malignant B-cell clone from a patient with follicular lymphoma. This self-antigenic recognition activates BCR-mediated signaling and provides survival signals to tumor cells [80].

## 5. Conclusion

Although initially discovered in skeletal muscle, MYOF has been found to abound in many other tissues as well as various cancers. This membrane protein mediates receptor internalization and recycling, endocytosis, exocytosis, and intercellular membrane structures. It is also associated with poor prognosis in many malignant diseases clinically. MYOF serves as an oncogenic protein implicated in various processes of tumor progression, including tumor cell proliferation, migration, invasion, EMT, angiogenesis, and metastasis (Table 1). Targeting MYOF with small interfering RNA and chemical molecules such as WJ460 has demonstrated significant antitumor effects in cancer, indicating that MYOF may be a potential target in the treatment of human cancer. We believe more compounds interfering with MYOF will be discovered, and further studies might be centred on the clinical use of such MYOF-targeting compounds.

## Figures and Tables

**Table 1:** Role of MYOF in specific tumor and its clinical significance.

Cancer type	Expression	Tumor biology	Clinical significance	Reference
Breast cancer	Increased	Increased cell proliferation, migration, invasion, EMT and metastasis	Poor outcome	[55, 56, 60, 65]
Pancreas cancer	Increased	Increased cell proliferation, migration and mitochondrial fitness	Poor prognosis and decreased overall survival	[48, 68, 70]
Lung cancer	Increased	Increased proliferation and angiogenesis	Poorer prognosis	[47, 77]
Liver cancer	Increased	Increased cell growth, invasion and proliferation	Not reported	[74]
Melanoma	Increased	Increased EMT, migration and invasion	Poor prognosis	[78]
Head and neck squamous cell carcinoma	Increased	Increased migration, metastasis	Poor overall survival, tumor recurrence, perineural invasion and distal metastasis	[50, 79]
Follicular lymphoma	Increased	Self-antigenic recognition, BCR-mediated signaling	The patient died	[80]
Endometrioid carcinoma	Decreased	No data	FIGO histologic grading score	[51]
Colon cancer	Increased	Mitochondrial fitness	Low patient survival	[72]
Renal cancer	Increased	No data	Poor patient prognosis. Predication of subsequent primary malignancy	[26, 81]

## References

[1] Lek A., Evesson F. J., Sutton R. B., North K. N., Cooper S. T. (2012). Ferlins: regulators of vesicle fusion for auditory neurotransmission, receptor trafficking and membrane repair. *Traffic*.

[2] Washington N. L. (2006). FER-1 regulates Ca^2+^-mediated membrane fusion during C. elegans spermatogenesis. *Journal of Cell Science*.

[3] Covian-Nares J. F., Koushik S. V., Puhl R. H. L., Vogel S. S. (2010). Membrane wounding triggers ATP release and dysferlin-mediated intercellular calcium signaling. *Journal of Cell Science*.

[4] Smith M. K., Wakimoto B. T. (2007). Complex regulation and multiple developmental functions of misfire, the Drosophila melanogaster ferlin gene. *BMC Developmental Biology*.

[5] Yasunaga S., Grati M., Cohen-Salmon M. (1999). A mutation in OTOF, encoding otoferlin, a FER-1-like protein, causes DFNB9, a nonsyndromic form of deafness. *Nature Genetics*.

[6] Bansal D., Miyake K., Vogel S. S. (2003). Defective membrane repair in dysferlin-deficient muscular dystrophy. *Nature*.

[7] Davis D. B., Delmonte A. J., Ly C. T., McNally E. M. (2000). Myoferlin, a candidate gene and potential modifier of muscular dystrophy. *Human Molecular Genetics*.

[8] Fardeau M., Brown R. H., de Jong P. J. (1998). Dysferlin, a novel skeletal muscle gene, is mutated in Miyoshi myopathy and limb girdle muscular dystrophy. *Nature Genetics*.

[9] Anderson L. V., Davison K., Moss J. A. (1999). Dysferlin is a plasma membrane protein and is expressed early in human development. *Human Molecular Genetics*.

[10] Doherty K. R., Cave A., Davis D. B. (2005). Normal myoblast fusion requires myoferlin. *Development*.

[11] de Morrée A., Hensbergen P. J., van Haagen H. H. H. B. M. (2010). Proteomic analysis of the Dysferlin protein complex unveils its importance for sarcolemmal maintenance and integrity. *PLoS One*.

[12] Demonbreun A. R., Rossi A. E., Alvarez M. G. (2014). Dysferlin and Myoferlin regulate transverse tubule formation and glycerol sensitivity. *The American Journal of Pathology*.

[13] Lostal W., Bartoli M., Roudaut C. (2012). Lack of correlation between outcomes of membrane repair assay and correction of dystrophic changes in experimental therapeutic strategy in dysferlinopathy. *PloS One*.

[14] Davis D. B., Doherty K. R., Delmonte A. J., McNally E. M. (2002). Calcium-sensitive phospholipid binding properties of normal and mutant ferlin C2 domains. *Journal of Biological Chemistry*.

[15] Vainzof M., Anderson L. V. B., McNally E. M. (2001). Dysferlin protein analysis in limb-girdle muscular dystrophies. *Journal of Molecular Neuroscience*.

[16] Inoue M., Wakayama Y., Kojima H. (2006). Expression of Myoferlin in skeletal muscles of patients with dysferlinopathy. *The Tohoku Journal of Experimental Medicine*.

[17] Leung C., Shaheen F., Bernatchez P., Hackett T.-L. (2012). Expression of myoferlin in human airway epithelium and its role in cell adhesion and zonula occludens-1 expression. *PloS One*.

[18] Robinson J. M., Vandré D. D., Ackerman W. E. (2009). Placental proteomics: a shortcut to biological insight. *Placenta*.

[19] Robinson J. M., Ackerman W. E., Behrendt N. J., Vandre D. D. (2009). While Dysferlin and Myoferlin are coexpressed in the human placenta, only Dysferlin expression is responsive to trophoblast fusion in model systems1. *Biology of Reproduction*.

[20] Bernatchez P. N., Acevedo L., Fernandez-Hernando C. (2007). Myoferlin regulates vascular endothelial growth factor receptor-2 stability and function. *Journal of Biological Chemistry*.

[21] Iacobuzio-Donahue C. A., Maitra A., Olsen M. (2003). Exploration of global gene expression patterns in pancreatic adenocarcinoma using cDNA microarrays. *The American Journal of Pathology*.

[22] Turtoi A., Musmeci D., Wang Y. (2011). Identification of novel accessible proteins bearing diagnostic and therapeutic potential in human pancreatic ductal adenocarcinoma. *Journal of Proteome Research*.

[23] Skubitz A. P. N., Pambuccian S. E., Argenta P. A., Skubitz K. M. (2006). Differential gene expression identifies subgroups of ovarian carcinoma. *Translational Research*.

[24] Kristiansen G., Pilarsky C., Wissmann C. (2005). Expression profiling of microdissected matched prostate cancer samples reveals CD166/MEMD and CD24 as new prognostic markers for patient survival. *The Journal of Pathology*.

[25] Amatschek S., Koenig U., Auer H. (2004). Tissue-wide expression profiling using cDNA subtraction and microarrays to identify tumor-specific genes. *Cancer Research*.

[26] Song D. H., Ko G. H., Lee J. H. (2017). Prognostic role of myoferlin expression in patients with clear cell renal cell carcinoma. *Oncotarget*.

[27] Shin O.-H., Han W., Wang Y., Südhof T. C. (2005). Evolutionarily conserved multiple C2 domain proteins with two transmembrane regions (MCTPs) and unusual Ca^2+^ binding properties. *Journal of Biological Chemistry*.

[28] Marty N. J., Holman C. L., Abdullah N., Johnson C. P. (2013). The C2 domains of Otoferlin, Dysferlin, and Myoferlin alter the packing of lipid bilayers. *Biochemistry*.

[29] Sudhof T. C., Rizo J. (1996). Synaptotagmins: C2-domain proteins that regulate membrane traffic. *Neuron*.

[30] Harsini F. M., Bui A. A., Rice A. M. (2019). Structural basis for the distinct membrane binding activity of the homologous C2A domains of Myoferlin and Dysferlin. *Journal of Molecular Biology*.

[31] Harsini F. M., Chebrolu S., Fuson K. L., White M. A., Rice A. M., Sutton R. B. (2018). FerA is a membrane-associating four-helix bundle domain in the ferlin family of membrane-fusion proteins. *Scientific Reports*.

[32] Patel P., Harris R., Geddes S. M. (2008). Solution structure of the inner DysF domain of Myoferlin and implications for limb girdle muscular dystrophy type 2B. *Journal of Molecular Biology*.

[33] Redpath G. M. I., Sophocleous R. A., Turnbull L., Whitchurch C. B., Cooper S. T. (2016). Ferlins show tissue-specific expression and segregate as plasma membrane/late endosomal or trans-golgi/recycling ferlins. *Traffic*.

[34] Doherty K. R., Demonbreun A. R., Wallace G. Q. (2008). The endocytic recycling protein EHD2 interacts with Myoferlin to regulate myoblast fusion. *Journal of Biological Chemistry*.

[35] Naslavsky N., Caplan S. (2011). EHD proteins: key conductors of endocytic transport. *Trends in Cell Biology*.

[36] Rapaport D., Auerbach W., Naslavsky N. (2006). Recycling to the plasma membrane is delayed in EHD1 knockout mice. *Traffic*.

[37] Lenhart K. C., Becherer A. L., Li J. (2014). GRAF1 promotes ferlin-dependent myoblast fusion. *Developmental Biology*.

[38] Posey J. A. D., Pytel P., Gardikiotes K. (2011). Endocytic recycling proteins EHD1 and EHD2 interact with fer-1-like-5 (Fer1L5) and mediate myoblast fusion. *The Journal of Biological Chemistry*.

[39] Demonbreun A. R., Posey A. D., Heretis K. (2010). Myoferlin is required for insulin-like growth factor response and muscle growth. *FASEB Journal : Official Publication of the Federation of American Societies for Experimental Biology*.

[40] Yu C., Sharma A., Trane A., Utokaparch S., Leung C., Bernatchez P. (2011). Myoferlin gene silencing decreases Tie-2 expression in vitro and angiogenesis in vivo. *Vascular Pharmacology*.

[41] Bernatchez P. N., Sharma A., Kodaman P., Sessa W. C. (2009). Myoferlin is critical for endocytosis in endothelial cells. *American Journal of Physiology-Cell Physiology*.

[42] Cipta S., Patel H. H. (2009). Molecular bandages: inside-out, outside-in repair of cellular membranes. Focus on ‘Myoferlin is critical for endocytosis in endothelial cells’. *American Journal of Physiology-Cell Physiology*.

[43] Miyatake Y., Yamano T., Hanayama R. (2018). Myoferlin-mediated lysosomal exocytosis regulates cytotoxicity by phagocytes. *The Journal of Immunology*.

[44] Schiller C., Diakopoulos K. N., Rohwedder I. (2013). LST1 promotes the assembly of a molecular machinery responsible for tunneling nanotube formation. *Journal of Cell Science*.

[45] Chauveau A., Aucher A., Eissmann P., Vivier E., Davis D. M. (2010). Membrane nanotubes facilitate long-distance interactions between natural killer cells and target cells. *Proceedings of the National Academy of Sciences*.

[46] Blomme A., Fahmy K., Peulen O. (2016). Myoferlin is a novel exosomal protein and functional regulator of cancer-derived exosomes. *Oncotarget*.

[47] Song D. H., Ko G. H., Lee J. H. (2016). Myoferlin expression in non-small cell lung cancer: Prognostic role and correlation with VEGFR-2 expression. *Oncology Letters*.

[48] Wang W.-S., Liu X.-H., Liu L.-X. (2013). iTRAQ-based quantitative proteomics reveals myoferlin as a novel prognostic predictor in pancreatic adenocarcinoma. *Journal of Proteomics*.

[49] Sun Z., Wang L., Eckloff B. W. (2014). Conserved recurrent gene mutations correlate with pathway deregulation and clinical outcomes of lung adenocarcinoma in never-smokers. *BMC Medical Genomics*.

[50] Kumar B., Brown N. V., Swanson B. J. (2016). High expression of myoferlin is associated with poor outcome in oropharyngeal squamous cell carcinoma patients and is inversely associated with HPV-status. *Oncotarget*.

[51] Kim M. H., Song D. H., Ko G. H. (2018). Myoferlin expression and its correlation with FIGO histologic grading in early-stage endometrioid carcinoma. *Journal of Pathology and Translational Medicine*.

[52] Bray F., Ferlay J., Soerjomataram I., Siegel R. L., Torre L. A., Jemal A. (2018). Global cancer statistics 2018: GLOBOCAN estimates of incidence and mortality worldwide for 36 cancers in 185 countries. *CA: A Cancer Journal for Clinicians*.

[53] Webb D. J., Donais K., Whitmore L. A. (2004). FAK-CSrc signalling through paxillin, ERK and MLCK regulates adhesion disassembly. *Nature Cell Biology*.

[54] Wang Y., McNiven M. A. (2012). Invasive matrix degradation at focal adhesions occurs via protease recruitment by a FAK-Cp130Cas complex. *The Journal of Cell Biology*.

[55] Blackstone B. N., Li R., Ackerman W. E., Ghadiali S. N., Powell H. M., Kniss D. A. (2015). Myoferlin depletion elevates focal adhesion kinase and paxillin phosphorylation and enhances cell-matrix adhesion in breast cancer cells. *American Journal of Physiology-Cell Physiology*.

[56] Turtoi A., Blomme A., Bellahcene A. (2013). Myoferlin is a key regulator of EGFR activity in breast cancer. *Cancer Research*.

[57] Rush J. S., Quinalty L. M., Engelman L., Sherry D. M., Ceresa B. P. (2011). Endosomal accumulation of the activated epidermal growth factor receptor (EGFR) induces apoptosis. *Journal of Biological Chemistry*.

[58] Volakis L. I., Li R., Ackerman T. W. E. (2014). Loss of myoferlin redirects breast cancer cell motility towards collective migration. *PloS One*.

[59] Li R., Ackerman T. W. E., Mihai C., Volakis L. I., Ghadiali S., Kniss D. A. (2012). Myoferlin depletion in breast cancer cells promotes mesenchymal to epithelial shape change and stalls invasion. *PloS One*.

[60] Eisenberg M. C., Kim Y., Li R., Ackerman W. E., Kniss D. A., Friedman A. (2011). Mechanistic modeling of the effects of myoferlin on tumor cell invasion. *Proceedings of the National Academy of Sciences*.

[61] Lamouille S., Xu J., Derynck R. (2014). Molecular mechanisms of epithelial¨Cmesenchymal transition. *Nature Reviews Molecular Cell Biology*.

[62] Zhou Y., Xiong L., Zhang Y., Yu R., Jiang X., Xu G. (2016). Quantitative proteomics identifies myoferlin as a novel regulator of A Disintegrin and Metalloproteinase 12 in HeLa cells. *Journal of Proteomics*.

[63] Zhang T., Li J., He Y. (2018). A small molecule targeting myoferlin exerts promising anti-tumor effects on breast cancer. *Nature Communications*.

[64] Li Y., He Y., Shao T. (2019). Modification and biological evaluation of a series of 1,5-Diaryl-1,2,4-triazole compounds as novel agents against pancreatic cancer metastasis through targeting myoferlin. *Journal of Medicinal Chemistry*.

[65] Blomme A., Costanza B., de Tullio P. (2017). Myoferlin regulates cellular lipid metabolism and promotes metastases in triple-negative breast cancer. *Oncogene*.

[66] Pavlova N. N., Thompson C. B. (2016). The emerging hallmarks of cancer metabolism. *Cell Metabolism*.

[67] LeBleu V. S., O Connell J. T., Gonzalez Herrera K. N. (2014). PGC-1α mediates mitochondrial biogenesis and oxidative phosphorylation in cancer cells to promote? metastasis. *Nature Cell Biology*.

[68] Fahmy K., Gonzalez A., Arafa M. (2016). Myoferlin plays a key role in VEGFA secretion and impacts tumor-associated angiogenesis in human pancreas cancer. *International Journal of Cancer*.

[69] Rademaker G., Costanza B., Anania S. (2019). Myoferlin contributes to the metastatic phenotype of pancreatic cancer cells by enhancing their migratory capacity through the control of oxidative phosphorylation. *Cancers (Basel)*.

[70] Rademaker G., Hennequiére V., Brohée L. (2018). Myoferlin controls mitochondrial structure and activity in pancreatic ductal adenocarcinoma, and affects tumor aggressiveness. *Oncogene*.

[71] Bykov V. J. N., Lambert J. M. R., Hainaut P., Wiman K. G. (2009). Mutant p53 rescue and modulation of p53 redox state. *Cell Cycle*.

[72] Rademaker G., Costanza B., Bellier J. (2019). Human colon cancer cells highly express myoferlin to maintain a fit mitochondrial network and escape p53-driven apoptosis. *Oncogenesis*.

[73] Demonbreun A. R., Lapidos K. A., Heretis K. (2010). Myoferlin regulation by NFAT in muscle injury, regeneration and repair. *Journal of Cell Science*.

[74] Hermanns C., Hampl V., Holzer K. (2017). The novel MKL target gene myoferlin modulates expansion and senescence of hepatocellular carcinoma. *Oncogene*.

[75] Hampl V., Martin C., Aigner A. (2013). Depletion of the transcriptional coactivators megakaryoblastic leukaemia 1 and 2 abolishes hepatocellular carcinoma xenograft growth by inducing oncogene-induced senescence. *EMBO Molecular Medicine*.

[76] Ohrnberger S., Thavamani A., Braeuning A. (2015). Dysregulated serum response factor triggers formation of hepatocellular carcinoma. *Hepatology*.

[77] Leung C., Yu C., Lin M. I., Tognon C., Bernatchez P. (2013). Expression of Myoferlin in human and murine carcinoma tumors. *The American Journal of Pathology*.

[78] Zhang W., Zhou P., Meng A., Zhang R., Zhou Y. (2018). Down-regulating Myoferlin inhibits the vasculogenic mimicry of?melanomavia decreasing MMP-2 and inducing mesenchymal-to-epithelial transition. *Journal of Cellular and Molecular Medicine*.

[79] Yadav A., Kumar B., Lang J. C., Teknos T. N., Kumar P. (2017). A muscle-specific protein 'myoferlin' modulates IL-6/STAT3 signaling by chaperoning activated STAT3 to nucleus. *Oncogene*.

[80] Sachen K. L., Strohman M. J., Singletary J. (2012). Self-antigen recognition by follicular lymphoma B-cell receptors. *Blood*.

[81] Koh H. M., An H. J., Ko G. H. (2019). Identification of Myoferlin expression for prediction of subsequent primary malignancy in patients with clear cell renal cell carcinoma. *InVivo*.

